# Levels of Octachlorostyrene in Mothers’ Milk and Potential Exposure Among Infants in Sendai City, Japan 2012

**DOI:** 10.3390/ijerph17093064

**Published:** 2020-04-28

**Authors:** Sani Rachman Soleman, Tomoko Fujitani, Yukiko Fujii, Kouji H. Harada

**Affiliations:** 1 Department of Health and Environmental Sciences, Kyoto University Graduate School of Medicine, Kyoto 606−8501, Japan; sani.soleman.67s@st.kyoto-u.ac.jp (S.R.S.); fujitani.tomoko.4w@kyoto-u.ac.jp (T.F.); 2 Faculty of Medicine, Universitas Islam Indonesia, Yogyakarta 55584, Indonesia; 3 Department of Pharmaceutical Sciences, Daiichi University of Pharmacy, Fukuoka 815−8511, Japan; yu-fujii@umin.ac.jp

**Keywords:** octachlorostyrene, lipid content, mother’s milk, persistent organic pollutants

## Abstract

Persistent organic pollutants can accumulate inside the human body, including in mothers’ milk, which may affect infant development. This cross-sectional study aimed to examine selected persistent organic pollutants in the milk of 100 mothers in Sendai city, Miyagi Prefecture, Japan. We used gas-chromatography-electron capture negative chemical ionization-mass spectrometry to check for octachlorostyrene, dechlorane (Dec) plus, Dec 602, Dec 603, and Dec 604. Octachlorostyrene was detected in 86 samples at more than the method detection limit (84 pg g-lipid^−1^) but no dechloranes were above the method detection limit (1 ng mL^−1^ for dechlorane plus, Dec 602, and Dec 603; 20 ng mL^−1^ for Dec 604). The mean octachlorostyrene concentration was 461 pg g-lipid^−1^, the median was 337 pg g-lipid^−1^, and the standard deviation 450 pg g-lipid^−1^. No baseline characteristics were associated with octachlorostyrene level except for mother’s occupation (stay-at-home mother, 353 ± 327 pg g-lipid^−1^; others, 531 ± 509 pg g-lipid^−1^). Octachlorostyrene was also significantly negatively correlated with lipid content (r = −0.35, *p* = 0.0004). However, the maximum intake of octachlorostyrene among infants in this study (3.5 ng/kg/day) was under the acceptable daily intake (30 ng/kg/day, derived from 12−month study in rats), and is therefore unlikely to pose a health risk.

## 1. Introduction

Harmful substances have been used in Japan and can impact the environment and pose human health hazards [1]. Persistent organic pollutants (POPs) are organic chemical compounds that accumulate in lipid metabolism systems, disrupting hormonal processes. Particularly harmful are the so-called endocrine-disrupting chemicals [2]. Exposure to POPs has been widely evaluated [2]. 

Octachlorostyrene (OCS) is a persistent and bioaccumulative toxicant with halogenated aromatic components. Non-oncogenic effects of OCS have been found in liver cells via oxidative stresses [3,4]. OCS has also been reported to induce metabolic activation via an aryl hydrocarbon receptor and constitutive androstane receptor, and therefore could be a potential endocrine disruptor [5]. It is a primary level 1 chemical, as categorized by the Canada Ontario Agreement on Great Lake Water Quality and Ecosystem Health (COA) in 2014 [3]. Level 1 chemicals are persistent, bioaccumulative, toxic and of immediate environmental concern to the Great Lake Basin. OCS is created as a by-product of industrial manufacturing processes involving chlorine. It has never been commercially used, but is widely distributed in the environment. In industrial manufacturing, OCS originates from production of magnesium and chlorinated solvents, aluminum plasma etching, aluminum degassing with hexachloroethane, chlorination of titanium, and waste incineration. Environmental concern about chlorinated aromatic compounds is widespread [6], but there is little information available about how these are contaminated with OCS and released to the environment. OCS has been found in several media including sediment, fish, marine products, and human blood [7]. Sediment samples from Calcasieu Lake in the USA showed 360 ng/g of OCS, while those from other lakes were around 0.2 ng/g [8]. One study found high concentrations in aquatic organisms (mean 0.06 ng/g in zebra mussels from Europe; mean 0.53 ng/g in cod liver oil in the United Kingdom) [7]. Fish consumption might be one exposure sources to OCS. Other studies have reported OCS in human blood (54.6 ng/g lipid in aluminum foundry workers in Sweden) and breast milk (0.18 ng/g lipid in healthy mothers from Denmark and Finland) [9,10]. 

Other emerging halogenated compounds are chlorinated flame retardants such as dechloranes (Decs). Decs were developed as substitutes for Mirex that were already regulated as POPs, and Decs are also considered as a persistent substance. Environmental contamination in aquatic systems by Decs has been reported [11]. Among several Decs, dechlorane plus (DP) was included in the Candidate List of substances of very high concern for authorization [12]. It was detected in human breast milk samples along with OCS in several cities [13,14]. Few studies are available on the toxicities, but expression of metabolic enzymes was induced [15]. OCS’s structural similarity to Mirex warrants further investigation.

These lipophilic compounds are accumulative in adipose tissues and breast milk. There is little information on internal burdens of OCS in humans. Exposure levels in humans needs investigation. In addition, factors affecting the exposure to OCS will be useful to reduce the exposure. Hence, the objective of this study was to examine OCS and Decs in breast milk of Japan, in Miyagi prefecture, and estimate exposure to detected OCS among infants. This study may help to assess the potential risk among an affected or vulnerable population. Moreover, the levels of detected OCS were compared with participant’s characteristics such as age, occupation, and fish intake to explore the related factors. 

## 2. Materials and Methods 

### 2.1. Study Participants

Breast milk samples were collected from mothers who gave birth at a general hospital in Sendai City, Miyagi Prefecture from June to December, 2012. They are a part of the samples in the Kyoto Human Specimen Bank, which uses a standardized protocol [16]. Briefly, approximately 300 g of breast milk were collected from lactating women within 2 months after delivery (average 326 g ranging from 93 g to 400 g). The hospital reported 1229 deliveries in 2012, and 100 samples were used for chemical analysis of environmental pollutants. Mothers were also asked to answer a questionnaire on their characteristics. Written consent was obtained from all mothers. The protocol for this study was approved by the Ethics Committee of Kyoto University Graduate School of Medicine and Faculty of Medicine and Hospital (Approval number E25 on Feb 1st, 2010, ‘Human exposure monitoring and risk assessment’). Samples were analyzed by gas-chromatography-electron capture negative chemical ionization-mass spectrometry (GC-ECNI-MS) to detect substances that had not previously been monitored, and to identify the current state of environmental pollution. 

### 2.2. Chemicals

We looked at levels of OCS, dechlorane plus (DP), Dec 602, Dec 603, and Dec 604. Syn-DP, anti-DP, and OCS (ULM−4559) were purchased from Cambridge Isotope Laboratories (Tewksbury, MA). Dec 602 (95%), Dec 603 (98%), and Dec 604 (98%) were purchased from Toronto Research Chemical Inc. (Toronto, ON, Canada). ^13^C_6_- hexachlorobenzene (manufactured by CIL) was used as a recovery surrogate substance. ^13^C_12_−2,3,3′, 5,5′-pentachlorobiphenyl (CB−111, manufactured by CIL) was used as an internal standard for quantification. Isopropanol, diethyl ether, hexane, nonane, and dichloromethane were used for pesticide residue tests and polychlorinated biphenyl tests (Kanto Chemical Co., Ltd., Tokyo, Japan). The Florisil was made by Wako Pure Chemical (Osaka, Japan).

### 2.3. Extraction and Purification of Samples

The milk sample was stirred, and 5 mL was separated into a polypropylene centrifuge tube. Then, 9 mL extraction solvent (2:1:3 (vol/vol/vol) isopropanol/diethyl ether/hexane), with a carbon 13-labeled hexachlorobenzene (500 pg) was added to the sample as a recovery standard, and samples were vortexed and centrifuged. The organic layer was transferred to a flask, and the extraction operation was repeated by adding another 8 mL of extraction solvent. The combined organic layers were concentrated using a rotary evaporator. The crude extract was diluted in 10 mL of hexane using a volumetric flask. An aliquot was divided, and the lipid weight was weighed. Distilled water was added to the crude extract, vortexed, centrifuged, and the aqueous layer was removed. Then, 10 mL of the crude extract was added dropwise to an 8 g activated Florisil column (Florisil PR, manufactured by Wako Pure Chemical Industries). Target analytes were eluted with 20 mL of hexane (first fraction containing OCS) with the loading solution, and 40 mL of a 10% dichloromethane/hexane solution (second fraction containing Dec). The eluate was concentrated to about 1 mL using a rotary evaporator. After concentrating to 0.1 mL of nonane, internal standard ^13^C_12_-labeled CB−111 (10 ng) was added and the sample was subjected to GC/MS analysis. 

### 2.4. Chemical Analysis 

Agilent 6890GC/5973MS was used. The capillary column was made of HP−5MS and had a total length of 30 m, an inner diameter of 0.25 mm, and a film thickness of 0.25 µm. The carrier gas was helium (99.9999% pure; Air Liquide Japan, Tokyo, Japan) with flow rate 1 mL min^−1^. The injection volume was 1.0 µL. The injector was used in splitless mode, with the vent opened at 1 min. The injector temperature was 280 °C. The oven temperature program started at 70 °C for 1.5 min, then increased at 20 °C min^−1^ to 230 °C, then at 4 °C min^−1^ to 280 °C. Substances were measured by negative ion chemical ionization using methane gas. The ion source, triple-quadrupole, and transfer line temperatures were 150, 100, and 280 °C, respectively. The m/z 380 was used for quantification of OCS. DP, Dec 602, Dec 603, and Dec 604 were quantified using m/z 654, 614, 638, and 614, respectively. ^13^C_12_-labeled CB−111, hexachlorobenzene, and DP were monitored by m/z 340, 290, and 664, respectively. The instrumental limit of detection (IDL) was defined as a S/N ratio of 3. Using a blank sample, contamination in the extraction and purification was evaluated. Signals were below IDL in the blank sample, so the method detection limit (MDL) was equal to the IDL. Recoveries of standard-spiked samples (200 pg for OCS, 20 ng for DP, Dec 602, and Dec 603, and 500 ng for Dec 604, *n* = 5) were 96% for OCS and 92%–98% for Dec.

### 2.5. Statistical Analysis

Levels less than the MDL were converted to half of the MDL. The normality of the data was tested using the Kolmogorov–Smirnov method. An independent *t*-test was used to examine the differences in OCS levels by baseline characteristics. Multiple regression analyses were used to examine correlations between OCS and maternal and infant characteristics. SPSS Statistics 23 (IBM, Chicago, IL, USA) was used for these calculations.

## 3. Results and Discussion 

Among 100 samples, 98 mothers responded to the questionnaire. Maternal characteristics are summarized in Table 1. The mothers’ mean age was 30 years old. The biggest groups in each category were stay-at-home mothers (36%), nulliparous (60%), vaginal delivery (57%), and never smoked (70%). Birth outcomes in this study were comparable to those in Japan [17]. There was no available data on fish intake among breast feeding mothers, but the levels of fish intake among pregnant women (29.1 g/day) was lower than current participants [18]. This could be due to a difference in the food frequency questionnaires.

OCS was detected in 86 samples at more than the MDL (84 pg g-lipid^−1^). OCS concentration had a mean of 461 pg g-lipid^−1^, median of 337, and standard deviation of 450 (Table 2). The distribution followed a log-normal distribution. The levels were slightly higher than those reported in Finland and Denmark [8], suggesting that Japanese mothers may have higher exposure to OCS.

No dechloranes were found above the MDL (1 ng mL^−1^ for DP, Dec 602, and Dec 603; 20 ng mL^−1^ for Dec 604) and so were excluded from further analyses. DP levels in the Japanese environment were reported [19,20], and they were shown to be comparable to or lower than those in other countries. In addition, milk/serum ratio of DP was less than 1 [21]. Hence, a large sample volume would be required to detect Decs.

The association between OCS and maternal characteristics is shown in Table 3. Participants were divided into two groups by status or median values. Those with occupations other than stay-at-home mother had higher OCS levels. This finding suggests that OCS levels in the home environment may be lower than in the workplace or that there may be occupational exposures to OCS. Since we have only two categories of occupation (stay-at-home mother and other), it is difficult to identify the distinct exposure sources in this study. As mentioned before, OCS can be a by-product in chemical processes, but the exposure routes in the general population should be investigated. Lipid content was negatively associated with OCS level (Pearson’s r = −0.35, *p* = 0.0004). Partition of OCS to breast milk could therefore be less fat-dependent.

Associations between OCS and infant characteristics are shown in Table 4. None of the variables showed any significant differences. 

Finally, multiple regression analysis was used to identify potential factors affecting OCS levels in breast milk. Table 5 shows the five models evaluated. Only lipid content of breast milk was negatively associated with OCS level (*p* value < 0.001). However, R^2^ was 9.8% and lipid content cannot fully explain the OCS levels. 

After ingestion of POPs into the gastrointestinal tract, they can be deposited in adipose tissue via lipoproteins, increasing the risk of cardiovascular disease [22]. A number of studies have clearly explained the association of POPs with alterations in lipid metabolism in both animals and people [23,24]. A study also demonstrated that prolonged exposure to polychlorinated biphenyls was correlated with lipid alteration [25]. This study found that OCS was significantly inversely correlated with lipid content of breast milk. The finding is similar to another study suggesting that organochlorine pesticides were inversely related to low density lipoprotein cholesterol levels in participants [23]. It is unclear whether OCS reduces the lipid content or a decrease in lipid content results in increased OCS concentration per lipid. If OCS causes changes in the lipid content of breast milk, it should be a concern to mothers, especially those breastfeeding. Toxicological investigation is required to explain the relationship. Moreover, the OCS exposure to fetuses may cause congenital disease [8]. Many researches have focused on the endocrine-disrupting effects of POPs, including OCS, affecting reproductive health in offspring, such as cryptorchidism [8].

We also calculated exposure to OCS among infants via breastfeeding. Consumption of breast milk was assumed to be 600 g/day and body weight of 1-year-old infants was set as 7.3 kg, in line with published figures [26]. 

The estimate of OCS intake is shown in Table 6. In this study population, the mean exposure of infants to OCS was 804 pg/kg/day, with a maximum of 3473 pg/kg/day. 

A report from New York State suggests that the acceptable daily intake (ADI) of OCS is 30 ng/kg/day [27]. Maximum intake in this study was therefore under this limit, and there is likely to be little risk to health. However, it should be noted that the estimate assumed constant levels of OCS during lactation. OCS concentrations in breast milk might change due to body weight change in mothers and elimination of OCS via breastfeeding. In addition, ADI was based on histological changes in liver, thyroid, and kidney in a 12-month dietary study in rats [28]. Even though ADI was calculated with an uncertainty factor of 1000, developmental effects would be considered in the population. Hence, the estimate has an uncertainty. Few epidemiological studies have examined the effects of OCS exposure on infant health. Further toxicological and epidemiological studies are required because mothers are still being exposed to OCS. 

In this study, factors other than occupation and lipid content showed no association with OCS levels in breast milk. This may be partly due to the characteristics of the participants. They were relatively healthy, and their birth outcomes and habits were within normal ranges. Comparisons between poor and normal outcomes might be required to show the possible association. On the other hand, OCS levels in this study were lower than other POPs (dichlorodiphenyltrichloroethane, 0.77−2.4 ng glipid^−1^; polychlorinated biphenyls, 129 ng glipid^−1^) in the same location [29,30]. The possible effects of OCS might be masked with those from other POPs. Available data on levels of OCS in human samples are scarce, especially for breast milk, and it is not clear if the infants outside Japan are at safe levels. This study analyzed breast milk samples collected in 2012. Significant temporal change in exposure is unlikely, but we recommend a prospective study to predict OCS exposure on birth outcome among infants. Finally, a comprehensive evaluation and monitoring OCS level with other POPs should be conducted to prevent health effects of OCS among breastfeeding mothers and their infants. 

## 4. Conclusions

OCS was detected in most breastmilk samples from Japanese mothers, and the levels were inversely correlated with lipid content. However, the estimated intake for infants in a city in Japan was lower than the current acceptable daily intake. 

## Figures and Tables

**Table 1:** Baseline characteristic of respondents at sampling of breast milk.

Characteristics	*n* (%)
**Occupation**	
Clerical workers	19 (19)
Stay-at-home mother	36 (36)
Medical practitioner	17 (17)
Service officer	25 (25)
NA	3 (3)
**Method of delivery**	
Cesarean	28 (28)
Suction	13 (13)
Vaginal	57 (57)
NA	2 (2)
**Sex of infants**	
Male	50 (50)
Female	48 (48)
NA	2 (2)
**Second-hand smokers**	
Husband	9 (9)
No	87 (87)
Parents	2 (2)
NA	2 (2)
**Parity**	
Nulliparous	58 (58)
Multiparous 1	29 (29)
2	8 (8)
3	3 (3)
NA	2 (2)
**Smoking**	
Never	69 (69)
Ex smoking	28 (28)
Current	1 (1)
NA	2 (2)
**Drinking alcohol**	
Never	29 (29)
Ex drinking	68 (56)
Current	0 (0)
NA	3 (2)
**Living near busy road**	
Yes	50 (50)
No	46 (46)
NA	4 (4)
	(mean ± SD)
**Age (yr)**	30 ± 6.6
**Height (cm)**	156 ± 22.9
**Weight (kg)**	54.9 ± 10.5
**Weight before pregnancy (kg)**	51.4 ± 10.3
**Birth weight (g)**	2993 ± 640
**Birth length (cm)**	45.5 ± 11.8
**Fish intake (g/day)**	45.3 ± 36.2

NA: not applicable due to missing records. Smokers (‘current’ and ‘ex smoking’) had 4.2 pack-years (SD 4.0). Ex-drinker of alcohol had 5.3 g-ethanol/d (SD 6.2).

**Table 2:** Distribution of octachlorostyrene (OCS) levels (pg g-lipid^−1^) among participants (*N* = 100).

	Mean	Median	SD	Min	Max
**OCS**	461	337	450	<84	3186

**Table 3:** Association between OCS and maternal characteristics at the collection of samples.

Characteristic	*n*	OCS (pg g-lipid^−1^) Mean ± SD	*p* value *
**Age at delivery**			
<32 yr	52	519 ± 551	0.50
≥32 yr	46	402 ± 306	
**Occupation**			
stay-at-home mother	36	353 ± 327	0.03 *
others	61	531 ± 509	
**Parity**			
nulliparous	60	478 ± 492	0.74
multiparous	40	445 ± 398	
**Delivery method**			
vaginal	57	513 ± 509	0.23
others	41	410 ± 370	
**Weight before pregnancy**			
<52 kg	54	472 ± 515	0.91
≥52 kg	44	462 ± 373	
**Age at first delivery**			
<29 yr	53	504 ± 538	0.59
≥29 yr	45	417 ± 330	
**Gestational week**			
<39 week	53	453 ± 355	0.7
≥39 week	42	454 ± 536	
**Fish intake**			
<40 g/day	49	482 ± 528	0.66
≥40 g/day	49	446 ± 371	
**Lipid content (%)**			
<2.34	51	594 ± 561	<0.01 *
≥2.34	49	322 ± 227	
**BMI**			
<22	49	460 ± 512	0.89
≥22	49	468 ± 394	
**Smoking**			
No	69	486 ± 490	0.43
Yes	29	413 ± 359	
**Drinking alcohol**			
No	29	458 ± 391	0.99
Yes	68	470 ± 484	
**Living near busy road**			
No	46	489 ± 537	0.64
Yes	50	422 ± 341	

* Independent *t*-test. Participants were divided into two groups by status or median values. Missing data in each variable were excluded from analysis.

**Table 4:** Association between OCS and infant characteristics.

Characteristic	*n*	OCS (pg g-lipid−1) Mean ± SD	*p* value *
**Sex of infants**			
**Female**	48	428 ± 357	0.43
**Male**	50	499 ± 533	
**Birth weight**			
**<3041 g**	49	469 ± 529	0.86
≥**3041 g**	49	460 ± 371	
**Birth length**			
**<49 cm**	56	436 ± 397	0.55
≥**49 cm**	38	481 ± 541	

* Independent *t*-test. Participants were divided into two groups by status or median values. Missing data in each variable were excluded from analysis.

**Table 5:** Multiple regression analysis of OCS level.

Model		Unstandardized Coefficients	Std. Error	Coefficients	*p* value *
**Model 1**	(constants)	87.564	53.232		0.10
	Weight before pregnancy	0.974	2.582	0.053	0.71
	Gestational week	−5.324	7.381	−0.076	0.47
	Fish intake	−0.139	0.378	−0.038	0.71
	Lipid contents	−91.190	26.582	−0.354	<0.001 *
	BMI	−5.796	4.496	−0.175	0.20
**Model 2**	(constants)	88.298	52.929		0.10
	Weight before pregnancy	0.750	2.497	0.041	0.76
	Gestational week	−5.500	7.329	−0.079	0.46
	Lipid contents	−90.209	26.316	−0.350	<0.001 *
	BMI	−5.599	4.442	−0.169	0.21
**Model 3**	(constants)	92.237	51.051		0.07
	Gestational week	−5.900	7.170	−0.084	0.41
	Lipid contents	−90.878	26.086	−0.353	<0.001 *
	BMI	−4.731	3.357	−0.143	0.16
**Model 4**	(constants)	52.450	16.241		0.002
	Lipid contents	−87.137	25.640	−0.338	<0.001 *
	BMI	−4.217	3.292	−0.127	0.20
**Model 5**	(constants)	32.296	4.043		<0.001
	Lipid contents	−84.669	25.658	−0.329	<0.001 *

Backward stepwise selection of variables was conducted. Variable with highest *p* value was eliminated from the model at each step. R^2^ = 9.8% for Model 5. * *p*-value is significant based on multiple regression analysis.

**Table 6:** Estimation of OCS intake among breastfed infants (1 year old).

OCS Intake (pg/kg/day)					
Mean	SD	Median	75th percentile	97.5th percentile	Max
804	624	722	1064	2766	3473
